# Supplementary material for: Evaluation of Antioxidant and Anticancer Activity of Mono- and Polyfloral Moroccan Bee Pollen by Characterizing Phenolic and Volatile Compounds
Source: Molecules. 2023 Jan 13;28(2):835. doi: 10.3390/molecules28020835 (PMC9866838; doi:10.3390/molecules28020835)
Supplement: Supplementary file 1 [file molecules-28-00835-s001.zip › molecules-2128179-supplementary.pdf]

Supplementary Material

Evaluation of Antioxidant and Anticancer Activity of Mono- and Polyfloral Moroccan Bee Pollen by Characterizing Phenolic and Volatile Compounds

Volkan Aylanc [1,2,3], Samar Larbi [1,2,4], Ricardo Calhelha [1,2], Lillian Barros [1,2], Feriel Rezouga [4], María Shantal Rodríguez-Flores [5], María Carmen Seijo [5], Asmae El Ghouizi [6], Badiaa Lyoussi [6], Soraia I. Falcão [1,2,*] and Miguel Vilas-Boas [1,2,*]

[1] Centro de Investigação de Montanha (CIMO), Instituto Politécnico de Bragança, Campus de Santa Apolónia, 5300-253 Bragança, Portugal
[2] Laboratório Associado para a Sustentabilidade e Tecnologia em Regiões de Montanha (SusTEC), Instituto Politécnico de Bragança, Campus de Santa Apolónia, 5300-253 Bragança, Portugal
[3] Departamento de Química e Bioquímica, Faculdade de Ciências, Universidade do Porto, 4169-007 Porto, Portugal
[4] Département de Génies Biologique et Agroalimentaire, Université Libre de Tunis, 30 Avenue Kheireddine Pacha, Tunis 1002, Tunisia
[5] Facultad de Ciencias, Universidad de Vigo, Campus As Lagoas, 36310 Vigo, Pontevedra, Spain
[6] Laboratory Physiology-Pharmacology and Environmental Health, Faculty of Sciences Dhar El Mehraz, University Sidi Mohamed Ben Abdallah, Fez 30050, Morocco
* Correspondence: sfalcao@ipb.pt (S.I.F.); mvboas@ipb.pt (M.V.-B.); Tel.: +351-273303401 (S.I.F.); +351-273303309 (M.V.-B.)

Table S1. Chromatographic and mass specifications for the phenolic and phenylamide observed in bee pollen.

Peak	t_R (min)	λ_{max} (nm)	$[M-H]^-$ *m/z*	MS^n (% base peak)	Proposed Compound
1	4.0	290, 322	341	MS^2:179 (100), 135 (2)	Caffeic acid hexoside[a,c]
2	4.0	292, 322	179	MS^2:179	Caffeic acid[a,b]
3	4.1	300	325	MS^2:163 (100)	*p*-coumaric acid hexoside[a]
4	6.7	259, 356	625	MS^2:317 (100)	Myricetin-3-*O*-rutinoside[a,d]
5	7.5	257, 353	625	MS^2:301 (100), 300 (90), 445 (82), 271 (15)	Quercetin-*O*-diglucoside[a,d]
6	8.1	258, 255	667	MS^2:316 (100), 317 (20), 625 (29)	Myricetin-*O*-acetyl deoxyhexosyl-hexoside[a]
7	8.4	267, 347	639	MS^2:315 (100), 477 (74), 300 (5); MS^3: 300 (100)	Methylherbacetin-*O*-dihexoside[a,e]
8	9.1	257, 357	479	MS^2:317 (100)	Myricetin-*O*-hexoside[a]
9	9.7	256, 352	595	MS^2:271 (11), 300 (100), 301 (56), 445 (37), 463 (25)	Quercetin-*O*-pentosyl-hexoside[a,f]
10	9.7	257, 354	609	MS^2:300 (100), 301(47)	Quercetin-3-*O*-rutinoside[a,d]
11	10.2	266, 348	609	MS^2:285 (95), 429 (100), 447 (12)	Kaempferol-*O*-diglucoside[a,g]
12	10.5	254, 353	623	MS^2:477 (100), 461 (50), 315 (11)	Isorhamnetin-*O*-deoxyhexosyl-*O*-hexoside[a,f]
13	10.5	259, 355	565	MS^2:521 (100); MS^3: 316 (100), 317 (46), 479 (21)	Myricetin-*O*-malonyl hexoside[a]
14	10.7	269, 347	623	MS^2:299 (58), 300 (37), 314 (100), 315 (70), 459 (89), 608 (32); MS^3: 299 (100)	Methylherbacetin-3-*O*-rutinoside[a,e]
15	11.0	266, 347	593	MS^2:285 (8), 431 (50), 447 (100)	Kaempferol-*O*-deoxyhexosyl-*O*-hexoside[a,h]

16	11.6	254, 354	609	MS^2: 315 (100)	Isorhamnetin-*O*-pentosyl-hexoside[a,d]
17	11.6	254, 354	609	MS^2: 315 (100)	Isorhamnetin-*O*-pentosyl-hexoside (isomer)[a,d]
18	11.6	310	163	MS^2: 118 (100)	*p*-coumaric acid[a,b]
19	11.8	256, 355	695	MS^2: 651 (100); MS^3: 609 (100), 300 (82), 301 (16)	Quercetin-*O*-malonyl deoxyhexosyl-hexoside[a]
20	12.1	267, 347	593	MS^2: 284 (100), 285 (69)	Kaempferol-3-*O*-rutinoside[a,e]
21	12.3	254, 354	623	MS^2: 314 (100), 315 (85), 459 (80)	Isorhamnetin-3-*O*-hexosyl-deoxyhexoside[a]
22	12.6	256, 354	463	MS^2: 301 (100)	Quercetin-3-*O*-glucoside[a,e]
23	12.6	253, 352	709	MS^2: 665 (100); MS^3: 314 (100), 315 (34)	Isorhamnetin-*O*-malonyl rutinoside[a,i]
24	13.0	255, 354	695	MS^2: 651 (100); MS^3: 315 (100), 300 (22)	Isorhamnetin-*O*-malonyl pentosyl-hexoside[a]
25	13.3	256, 353	549	MS^2: 505 (100); MS^3: 301 (100)	Quercetin-*O*-malonyl hexoside[a,i]
26	13.5	254, 359	579	MS^2: 535 (100); MS^3: 330 (34), 331 (100), 493 (12)	3',4',5',3,5,6,7-heptahydroxy-flavonol-*O*-malonyl hexoside[a]
27	13.9	254, 355	549	MS^2: 505 (100); MS^3: 301 (100)	Quercetin-*O*-malonyl hexoside (isomer)[a,i]
28	14.2	254, 347	447	MS^2: 301 (100)	Quercetin-3-*O*-rhamnoside[a,h]
29	14.3	253.351	477	MS^2: 314 (100), 315 (53)	Isorhamnetin-3-*O*-glucoside[a,d]
30	15.0	265, 347	533	MS^2: 489; MS^3: 285 (100)	Kaempferol-*O*-malonyl rutinoside[a]
31	15.4	254, 354	563	MS^2: 315 (100)	Isorhamnetin-*O*-malonyl hexoside[a,i]
32	16.2	264, 341	431	MS^2: 285 (100)	Kaempferol-3-*O*-rhamnoside[a,e]
33	16.3	256, 349	447	MS^2: 301 (100)	Quercetin-3-*O*-rhamnoside[a,d]
34	19.3	298, 308	614	MS^2: 494 (24), 478 (100), 452 (78), 358 (18)	N^1-*p*-coumaroyl-N^5, N^{10}-dicaffeoylspermidine[a,d,e]
35	21.2	290, 309	582	462 (100)	N^1, N^5, N^{10}-*tri*-*p*-coumaroylspermidine[a,d,e]
36	21.3	268, 347	285	MS^2: 285 (100), 257 (13), 151 (20)	Kaempferol[a,b]
37	22.0	353, 370	315	MS^2: 300 (100)	Isorhamnetin[a,b]
38	22.2	299, 310	598	MS^2: 462 (100), 478 (39), 452 (34), 342 (14)	N^1, N^5-*di*-*p*-coumaroyl-N^{10}-caffeoylspermidine[a,d]
39	22.8	295, 310	582	MS^2: 462 (100), 436 (9), 342(7)	N^1, N^5, N^{10}-*tri*-*p*-coumaroylspermidine (isomer)[a,d,e]
40	24.1	295, 310	582	MS^2: 462 (100), 436 (9), 342 (6)	N^1, N^5, N^{10}-*tri*-*p*-coumaroylspermidine (isomer)[a,d,e]
41	25.0	295, 310	582	MS^2: 462 (100), 436 (9), 342 (7)	N^1, N^5, N^{10}-*tri*-*p*-coumaroylspermidine (isomer)[a,d]
42	26.5	295, 310	582	MS^2: 462 (100), 436 (10), 342 (7)	N^1, N^5, N^{10}-*tri*-*p*-coumaroylspermidine (isomer)[a,d,e]
43	27.0	270	785	MS^2: 665 (100), 545 (14), 639 (13)	Tetracoumaroyl spermine[a,e]
44	28.7	280, 307	785	MS^2: 665 (100), 545 (14), 639 (13)	Tetracoumaroyl spermine (isomer)[a,e]
45	29.3	277, 310	785	MS^2: 665 (100), 545 (14), 639 (13)	Tetracoumaroyl spermine (isomer)[a,e]
46	30.3	289, 306	785	MS^2: 665 (100), 545 (14), 639 (13)	Tetracoumaroyl spermine (isomer)[a,e]
47	31.9	293, 310	785	MS^2: 665 (100), 545 (14), 639 (13)	Tetracoumaroyl spermine (isomer)[a,e]
48	34.0	299, 310	785	MS^2: 665 (100), 545 (14), 639 (13)	Tetracoumaroyl spermine (isomer)[a,e]

Confirmed with: [a] MS^n fragmentation; [b] Standard; References: [c] Kang et al. [1]; [d] El Ghouizi et al. [2]; [e] Aylanc et al. [3]; [f] Sobral et al. [4]; [g] Llorach et al. [5]; [h] Falcão et al. [6]; [i] Mihajlovic et al. [7]. BP: bee pollen.

Table S2. Retention time (Rt) and calculated LRI for volatile compounds found in Moroccan bee pollen samples.

Peak	Rt	Compound	LRI
1	2.6	2-propenylidene-cyclobutene	730
2	3.1	Hexanal	768
3	4.2	2-hexenal	830
4	5.4	Heptanal	883
5	5.8	2,5-dimethyl-pyrazine	901
6	6.9	1,2-cyclopentanedione	933
7	8.3	2,4-heptadienal	998
8	8.8	Ethyl hexanoate	999
9	8.9	Octanal	988
10	9.3	2,4-heptadienal (isomer)	998
11	9.6	Hexanoic acid	1010
12	9.9	Eucalyptol	1016
13	11.7	3,5-octadien-2-one	1057
14	11.9	2,6,6-trimethylbicyclo[3.1.1]hept-3-ylamine	1160
15	12.7	3,5-octadien-2-one (isomer)	1079
16	13.2	Nonanal	1091
17	13.2	Cis-β-terpineol	1093
18	14.1	Methyl octanoate	1113
19	15.1	Lilac aldehyde D	1138
20	15.4	2,6-nonadienal	1142
21	15.7	Isopinocarveol	1148
22	17.3	Octanoic acid	1184
23	17.5	Ethyl octanoate	1183
24	17.8	Lilac alcohol D	1195
25	18.1	β-cyclocitral	1201
26	18.6	Methyl 7-hexanoate	1212
27	18.7	Methyl nonanoate	1213
28	19.8	Anisaldehyde	1238
29	19.9	Geranyl vinyl ether	1240
30	21.1	3-cyclohex-1-enyl-prop-2-enal	1269
31	21.3	2-methyl-1-nonene-3-ine	1271
32	21.9	Ethyl nonanoate	1285
33	22.0	Nonanoic acid	1287
34	23.3	Methyl 8-methyl-nonanoate	1315
35	25.9	3-methyl-2-pent-2-enyl-cyclopent-2-enone	1376
36	26.3	Ethyl decanoate	1377
37	26.3	Methyl octanoate	1385
38	26.7	Caryophyllene	1394
39	27.4	Decanoic acid	1411

40	28.4	6,10-dimethyl-5,9-undecadien-2-one	1425
41	28.5	4,6-dimethyl-(Z)-5,9-undecadien-2-one	1435
42	29.6	β-ionone	1499
43	29.8	β-ionone epoxide	1466
44	31.8	10-methyl-methyl undecanoate	1516
45	34.8	Ethyl decanoate	1592
46	34.9	Ethyl dodecanoate	1594
47	36.0	5-(1-piperidyl)-furan-2-carboxaldehyde	1693

LRI: Linear retention index determined on a DB-5 MS fused silica column relative to a series of *n*-alkanes (C_7-C_{36}). Rt: Retention time; BP: bee pollen.

References

[1] Kang, J.; Price, W. E.; Ashton, J.; Tapsell, L. C.; Johnson, S. Identification and characterization of phenolic compounds in hydromethanolic extracts of sorghum wholegrains by LC-ESI-MSn. *Food Chem* **2016**, *211*, 215–226.

[2] el Ghouizi, A.; el Menyiy, N.; Falcão, S. I.; Vilas-Boas, M.; Lyoussi, B. Chemical composition, antioxidant activity, and diuretic effect of Moroccan fresh bee pollen in rats. *Vet World* **2020**, *13*, 1251.

[3] Aylanc, V.; Tomás, A.; Russo-Almeida, P.; Falcão, S. I.; Vilas-Boas, M. Assessment of bioactive compounds under simulated gastrointestinal digestion of bee pollen and bee bread: Bioaccessibility and antioxidant activity. *Antioxidants* **2021**, *10*, 651.

[4] Sobral, F.; Calhelha, R. C.; Barros, L.; Dueñas, M.; Tomás, A.; Santos-Buelga, C.; Vilas-Boas, M.; Ferreira, I. C. F. R. Flavonoid composition and antitumor activity of bee bread collected in northeast Portugal. *Molecules* **2017**, *22*, 248.

[5] Llorach, R.; Gil-Izquierdo, A.; Ferreres, F.; Tomás-Barberán, F. A. HPLC-DAD-MS/MS ESI Characterization of Unusual Highly Glycosylated Acylated Flavonoids from Cauliflower (*Brassica oleracea* L. var. *botrytis*) Agroindustrial Byproducts. *J Agric Food Chem* **2003**, *51*, 3895–3899.

[6] Falcão, S. I.; Vale, N.; Gomes, P.; Domingues, M. R. M.; Freire, C.; Cardoso, S. M.; Vilas-Boas, M. Phenolic profiling of Portuguese propolis by LC–MS spectrometry: Uncommon propolis rich in flavonoid glycosides. *Phytochem Anal* **2012**, *24*, 309–318.

[7] Mihajlovic, L.; Radosavljevic, J.; Burazer, L.; Smiljanic, K.; Velickovic, T. C. Composition of polyphenol and polyamide compounds in common ragweed (*Ambrosia artemisiifolia* L.) pollen and sub-pollen particles. *Phytochemistry* **2015**, *109*, 125–132.
